# Coxsackie B1 virus-like particle vaccine modified to exclude a highly conserved immunoreactive region from the capsid induces potent neutralizing antibodies and protects against infection in mice

**DOI:** 10.1186/s12929-025-01183-1

**Published:** 2025-09-08

**Authors:** Saana Soppela, Martín González-Rodríguez, Virginia M. Stone, Iiris Mustonen, Niila V. V. Jouppila, Vili Lampinen, Teemu Haikarainen, Malin Flodström-Tullberg, Ilkka S. Junttila, Minna M. Hankaniemi

**Affiliations:** 1 Virology and Vaccine Immunology, Faculty of Medicine and Health Technology, Tampere University, Tampere, Finland; 2 Cytokine Biology, Faculty of Medicine and Health Technology, Tampere University, Tampere, Finland; 3 Center for Infectious Medicine, Department of Medicine Huddinge, Karolinska Institutet, Karolinska University Hospital Huddinge, Stockholm, Sweden; 4 Structural Immunology, Faculty of Medicine and Health Technology, Tampere University, Tampere, Finland; 5 Fimlab Laboratories, Tampere, Finland; 6 Department of Clinical Microbiology, Fimlab Laboratories, Tampere, Finland; 7 Research Unit of Biomedicine, University of Oulu, Oulu, Finland; 8 Department of Clinical Microbiology, Nordlab, Oulu, Finland; 9 Faculty of Medicine and Health Technology, Tampere University, Tampere, Finland

**Keywords:** Enteroviruses, Coxsackie B1 virus, Virus-like particle, Vaccine, Immunogenicity, VP1 N-terminus, Neutralizing antibodies, Non-neutralizing antibodies

## Abstract

**Background:**

Enteroviruses, including Coxsackie B (CVB) viruses, can cause severe diseases such as myocarditis, pancreatitis, and meningitis. Vaccines can prevent these complications, but conserved non-neutralizing epitopes in the viral capsid may limit their effectiveness. The immunodominant PALXAXETG motif, located in the VP1 N-terminus, is a highly conserved region in enteroviruses that elicits non-neutralizing antibody responses. Virus-like particles (VLPs) offer a safe and effective vaccine platform because of their structural similarity to native viruses but lack viral genetic material. Importantly, VLPs can be structurally modified to exclude specific epitopes.

**Methods:**

Here, we produced a modified CVB1 virus-like particle (VLP) vaccine lacking 15 amino acids from the PALXAXETG motif (designated VLPΔpalxa) using the baculovirus-insect cell expression system. To confirm the structural integrity, we determined the crystal structure of the modified VLP with 3.2 Å resolution. We then conducted comprehensive immunogenicity studies in mice, including dose titration, comparison of two versus three immunizations, and post-vaccination viral challenge. In addition, we evaluated the impact of the AS04 adjuvant on the immunogenicity of unmodified and modified CVB1-VLP vaccines and the formalin-inactivated CVB1 vaccine.

**Results:**

The yield of CVB1-VLPΔpalxa was 29.5 mg/L, and the particles were shown to assemble similarly to unmodified CVB1-VLP. CVB1-VLPΔpalxa induced robust antibody responses, with neutralizing antibody titres comparable to or exceeding those elicited by unmodified VLP or inactivated virus vaccines. A 2 µg dose was identified as optimal, providing the highest neutralizing antibody titres. A third immunization significantly increased antibody levels, and all non-adjuvanted vaccines protected the mice from CVB1 challenge after the third dose. The addition of AS04 significantly enhanced the antibody response, particularly in both VLP groups.

**Conclusions:**

We demonstrated that with targeted structural modification of the CVB1-VLP capsid, immunodominant antibody responses against the conserved PALXAXETG motif can be avoided. We demonstrate that structural modification of CVB1-VLP is a viable strategy. Since the deleted epitope is known to be non-neutralizing, its deletion may help focus the immune response on more protective targets and thereby improve vaccine efficacy. The modified VLPs, particularly when adjuvanted, offer a promising approach for developing safe and effective enterovirus vaccines.

**Graphical Abstract:**

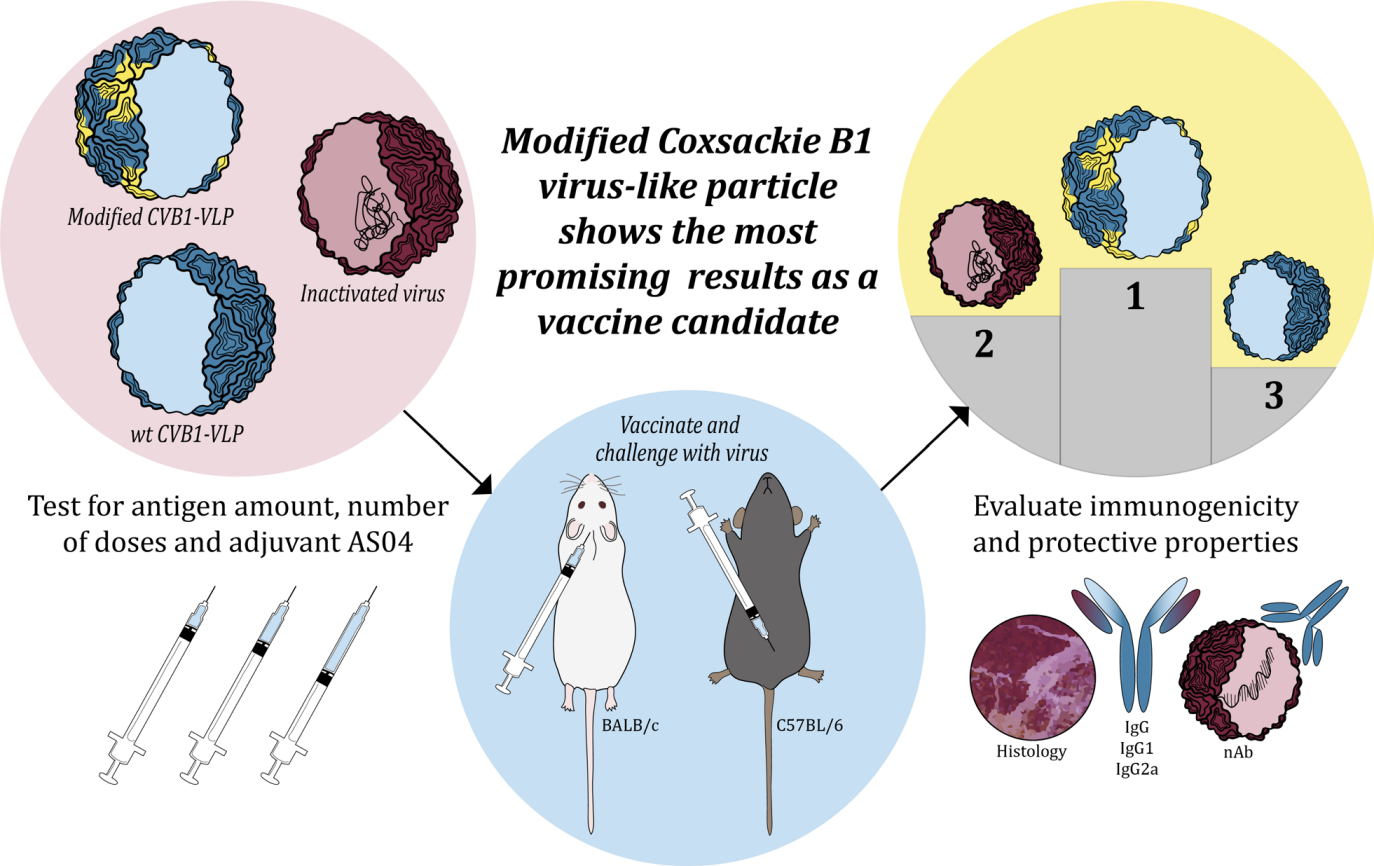

**Supplementary Information:**

The online version contains supplementary material available at 10.1186/s12929-025-01183-1.

## Background

Coxsackie B viruses (CVBs) belong to the Enterovirus (EV) B species of the *Picornaviridae* family and are divided into six serotypes (CVB1-CVB6). They are small, non-enveloped viruses with a positive-strand RNA genome of approximately 7.5 kb. The genome contains one open reading frame that is translated into a single polypeptide and further processed into structural and non-structural proteins. The domain that contains the structural proteins is first processed into the viral proteins VP0, VP1 and VP3 by a viral protease, and the VP0 domain is further processed to VP2 and VP4 by viral autocatalysis [1]. The structural proteins VP1-VP4 form protomers, and five of these protomers assemble into a pentamer. Finally, an icosahedral capsid, approximately 30 nm in diameter, consists of 12 pentamers. VP1, VP2, and VP3 are exposed on the particle surface, whereas VP4 is an internal protein [2, 3]. CVB infections commonly cause mild illnesses, such as common cold-like symptoms, but they can also lead to severe diseases, including meningitis [4], pancreatitis [5], and myocarditis [6, 7], particularly in immunocompromised individuals. Additionally, EV infections, particularly CVBs, may be linked to the development of type 1 diabetes (T1D) [8–10].

Virus-like particles (VLPs) are complex nanostructures that mimic the structure of infectious viruses but lack genetic material. These particles self-assemble from multiple proteins to replicate the conformation of authentic viruses [11, 12]. VLP-based vaccines, such as those for Human Papilloma Virus (HPV) [13, 14] and Hepatitis B [15], have demonstrated exceptional safety and efficacy, making VLP-vaccine technology an appealing approach for the prevention of EV infections. Traditional vaccine technologies have limitations, especially in terms of production efficacy and safety [16, 17]. However, VLP technology can be applied more broadly than traditional technologies, including with viruses that do not grow in cell cultures. Although VLPs structurally resemble viruses, they are incapable of replicating owing to the lack of a genetic code, making them safer than traditional vaccines.

A recent study revealed that individuals with T1D have predominantly non-neutralizing, CVB-reactive antibody responses following natural infection, whereas healthy individuals tend to have higher quantities of neutralizing antibodies [18]. In individuals at risk for T1D, serum samples showed a distinct pattern of anti-CVB antibody activity with non-neutralizing or infection enhancing responses whereas healthy individuals consistently had more neutralizing antibodies. Additionally, disease worsening following CVB3 infection has also been observed in mice with weak neutralizing antibody titres, leading to exacerbated, and sometimes lethal, myocarditis [19]. Picornavirus induced antibody-dependent infection enhancement, and the induction of type I interferons has been previously described more widely in vitro for foot-and-mouth disease virus [20], poliovirus [21], EV-A71 [22] and CVBs [18, 23]. High infectivity or disease enhancement may be induced when subneutralizing levels of antiviral antibodies or non-neutralizing antibodies facilitate viral entry into the host via Fc-receptor mediated endocytosis [24, 25]. These findings suggest that natural CVB infections may induce antibody responses that are not beneficial for the host, particularly in susceptible individuals. However, the specific epitopes responsible for these non-neutralizing or potentially enhancing responses remain unclear. Moreover, it is currently unknown whether inactivated whole-virus vaccines can also elicit such antibodies, as this has not been systematically studied.

Several studies have demonstrated that the N-terminal region of VP1 is a highly antigenic and immunoreactive region among enteroviruses. It contains several amino acids that are completely conserved in most enteroviruses [PALTA(A/V)E(I/T)G], with a core motif of approximately 9 amino acids (PALXAXETG-motif) [26–29]. This motif elicits robust and partially immunodominant antibody responses [26, 30], but antibodies directed against this region are not neutralizing, as expected for antibodies targeting conserved regions of the virus capsid. The monoclonal antibodies 5D-8.1 [31] and 3A6 [32] both recognize the N-terminal region of VP1 and are frequently used for the detection of enterovirus infections, demonstrating the immunodominance of this region. IPALTAVETGHT is the consensus sequence for the 5D-8.1 epitope [31], whereas 5D-8.1 and 3A6 both recognize the VP1 peptide RPTNSESIPALTAAE but not the overlapping downstream peptide PALTAVETGATNPLV [32].

A potential link between the PALXAXETG motif and disease worsening has been suggested after over 560 children were studied that suffered from acute flaccid myelitis (AFM) and motor paralysis during EV outbreaks in the U.S. [33]. Antibodies against this motif were detected in sera from 79% of AFM patients but not in those from 20% of non-AFM patients, suggesting a non-neutralizing character. Another study that focused on the N-terminus of Rhinovirus VP1 in mouse models suggested that the majority of VP1-specific antibody responses in individuals with repeated Rhinovirus infections are misdirected against a non-neutralizing epitope that is exposed during uncoating, suggesting this as a potential mechanism by which Rhinovirus might evade protective immunity in humans [34]. Additionally, another study linked the KEVPALTAVETGAT region to SS-A antibodies, which are associated with rheumatic autoimmune diseases, suggesting an association between PALXAXETG- and autoantibodies [35].

The main hypothesis tested in this study was that, through the exclusion of the highly conserved non-neutralizing and immunodominant region from the VP1 N-terminus of a VLP vaccine, the immune response could be directed toward more protective, neutralizing targets. A study by Mishra et al. (2019) demonstrated that the length of the PALXAXETG motif is 18 amino acids [33], whereas studies by Roivainen (1993) and Airaksinen (1999) have shown that the length of this region is 25 amino acids [27, 36]. On the basis of studies reporting the most immunoreactive parts of the PALXAXETG motif [27, 31–33, 36], we excluded the 15 amino acid-long region SESI**P**A**L**T**A**A**E**T**G**HT from the VP1 N-terminus of CVB1 and designated the deletion PALXA. Using this modified construct, we produced a VLP denoted VLPΔpalxa. P, L, A, E and G (in bold above) are the most conserved amino acids in the enterovirus PALXAXETG motif, the underlined part is the most conserved part of the motif.

While the N-terminus of VP1 is buried in the mature virion, EVs can spontaneously and reversibly externalize the N-terminus of VP1 in a process known as"breathing"[37]. This region also undergoes structural rearrangement and externalization during cell attachment [38]. However, the immunogenicity of the VP1 N-terminus does not appear to depend on its surface exposure, although it can momentarily become accessible in solution [36]. The immunogenicity of this buried region is likely due to internalization, processing and presentation of this peptide by antigen presenting cells, explaining the activation of B-cells. Furthermore, the non-neutralizing nature of antibodies targeting the PALXAXETG motif is most likely a result of its inaccessibility in the intact, mature virion, where this region remains buried and inaccessible for antibody binding. Importantly, while other buried epitopes may also be processed and elicit antibody responses, the PALXAXETG motif is the most frequently reported non-neutralizing epitope in enteroviruses and has been particularly associated with severe disease outcomes. Therefore, we selected this epitope for targeted deletion in our modified VLPs.

## Methods

### CVB1, CVB3 and CVB6 virus vaccines

Inactivated CVB1, CVB3 and CVB6 virus vaccines were used to study sequential immunization with different CVB serotypes. Inactivated CVB1 was based on strain 10796 (wild-type strain from Argentina) [39, 40] and was produced [40] and purified [41] as described. CVB3 Nancy strain (kindly provided by Dr. G. Frisk, Uppsala University) and the CVB6 reference strain (from ATCC) were produced and purified as described previously [42]. The viruses were inactivated with 0.01% (v/v) formalin for 5 days at 37 °C. Virus inactivation was confirmed by the lack of infectious virus (after culturing the inactivated viruses in GMK cells) in the median tissue culture infectious dose (TCID50) end-point dilution assay (detection limit, 49 TCID_50_ units/ml), as previously described [40].

### Coxsackie B virus-like particle design, production, and purification

Our previously published unmodified CVB1-VLP [40] (here termed Full VLP) was used in this study in addition to VLPΔpalxa, which was designed on the basis of the same sequence (GenBank accession number PP782006) but excludes the 15-amino acid region (SESIPALTAAETGHT) located at the N-terminal end of the CVB1 VP1 protein.

CVB1-VLPs were produced and purified according to a previously established protocol [43], including Tangential Flow Filtration (TFF) and chromatographic procedures. Briefly, the baculoviral transfer vector pOET5 (GenScript) contained separate expression cassettes for the CVB1 VP0-3 polyprotein (under the polyhedrin promoter) and the 3CD protease (under the CMV promoter). Recombinant baculoviruses were generated via the FlashBAC ULTRA baculovirus genome (Oxford Expression Technologies, UK) and amplified in Sf9 insect cells (Thermo Fisher Scientific).

CVB1-VLPs were expressed in High-Five insect cells at a multiplicity of infection (MOI) of 1. Five days post infection, the VLP-containing supernatant was harvested, clarified via Sartoclear Dynamics Lab filtration (Sartorius), and further processed via TFF with a 750 kDa MWCO hollow fibre in an ÄKTA Flux system (Cytiva). The buffer was exchanged with 40 mM Tris–HCl (pH 7.5), 10 mM MgCl_2_, and 40 mM NaCl during TFF.

Purification involved ion-exchange chromatography using HiLoad™ 26/10 Q Sepharose High Performance and HiTrap Q Sepharose XL columns (Cytiva) to remove impurities and the final concentration of the VLPs with TFF. The final VLP preparations were stored in Tris-buffered saline (40 mM Tris–HCl, pH 7.4, 10 mM MgCl_2_, 0.2 M NaCl + 0.1% Tween 80) at −80 °C.

### Virus-like particle characterization

The protein composition, structural integrity, and purity of the purified CVB1-VLPs were analyzed via multiple biochemical and biophysical methods. Total protein profiles were assessed with any kD stain-free precast gels (Bio-Rad, USA), followed by densitometric analysis with Image Lab software (Bio-Rad). Residual baculoviruses were detected with a mouse monoclonal anti-gp64 antibody (1:2000, Santa Cruz Biotechnology, USA), followed by an IRDye 800CW goat anti-mouse IgG secondary antibody (1:40,000, LI-COR Biosciences, USA).

To evaluate vaccine purity, residual DNA content was quantified using the Quant-iT dsDNA high-sensitivity kit (Thermo Fisher Scientific), whereas endotoxin levels were measured with the Pierce LAL Chromogenic Endotoxin Quantitation Kit (Thermo Fisher Scientific). Any remaining endotoxins were removed using the EndoTrap® HD FPLC column (Lionex GmbH, Germany) according to the manufacturer’s protocol.

The particle size distribution and homogeneity of the purified VLPs were analyzed with dynamic light scattering (DLS) via a Zetasizer Nano ZS (Malvern Instruments, UK). The analysis consisted of six consecutive measurements, each containing 15 readings at 10 s intervals. Morphological characterization was performed with transmission electron microscopy (TEM), where negatively stained VLPs (1% uranyl acetate) were imaged using a JEOL F200 S/TEM (Japan) at 80 kV.

The thermal stability of the VLPs was assessed via differential scanning calorimetry (DSC) under previously established conditions [44]. The measurements were conducted in 40 mM Tris–HCl + 10 mM MgCl_2_ + 0.2 M NaCl + 0.1% Tween 80 buffer at a final VLP concentration of 0.5 mg/ml.

### Protein crystallization

For more detailed structural analysis, VLPΔpalxa (4.8 mg/ml) was crystallized using a hanging-drop vapor-diffusion method. Equal volumes (1 μl) of well solution (0.1 M Na-acetate pH 6.5, 1.5 M Li_2_SO_4_) and protein solution were mixed and incubated at 22 °C. Before data collection, the crystals were cryo-protected with 25% glycerol in well solution and flash-frozen in liquid nitrogen.

### Data collection, structure determination, and refinement

Diffraction data were collected on the beam line I03 at Diamond Light Source, Didcot, UK. Data were processed and scaled with the XDS package [45] and the structure was determined with molecular replacement using the Coxsackievirus B3 structure (PDB ID: 1COV) as a search model. The refinement was carried out with phenix.refine [46] and Coot was used for model building [47]. Data collection and refinement statistics are shown in Supplementary Table S1.

### Safety, dose-titration and adjuvant testing in mice

All the animal experiments except for the challenge study were conducted at the Tampere University animal facility, Finland, in accordance with national and institutional ethical guidelines for animal care and use. Female BALB/cJRj and C57BL/6JRj mice (Janvier Labs) were housed in a specific pathogen-free environment in individually ventilated cages, with 3–5 mice per cage. Food and water were provided *ad libitum*. Before immunization, the mice underwent a one-week acclimatization period and were 5 weeks old at the time of the first dose.

All vaccines were formulated in PBS containing 0.1% Tween 80. To assess the effect of adjuvants, one immunization experiment included 5 µg of MPLA-SM VacciGrade (InvivoGen, USA) and 50 µg of Alhydrogel® adjuvant 2% (InvivoGen) per dose. Adjuvants were added to the vaccine preparation immediately before administration.

The mice were randomly assigned to groups and received subcutaneous injections of 150 µl of vaccine interscapularly. The primary immunization was administered at week 0, followed by booster doses at week 3 and, in some cases, week 6. The mice were euthanized in a CO_2_ chamber at week 6 or 9, and blood samples were collected into Microtainer SST Blood Collection Tubes (Becton Dickinson) for further analysis.

### CVB1 challenge study

C57Bl/6J female and male mice bred in-house were housed in a specific pathogen-free environment at the PKL animal facility, Karolinska University Hospital Huddinge, Sweden. Five mice per group were challenged with CVB1-10796 by intraperitoneal injection after receiving three doses of 2 µg of vaccine at three-week intervals. The virus was diluted in serum-free RPMI to a final concentration of 10^7^ PFU in a total volume of 200 μl. A blood sample was collected from the tail vein on day 3 post infection (1:1 dilution in 12 mmol EDTA-PBS). The mice were euthanized on day 5 post infection, after which blood was collected, and the pancreas and heart were harvested for histological analysis and viral titration.

Half of the pancreas tissues were formalin fixed, paraffin embedded, sectioned (5 µm), and stained with hematoxylin and eosin for histological evaluation. The remaining pancreas tissues were homogenized in Minimum Essential Medium Eagle (MEM) with Precellys ceramic beads (1.4 and 2.8 mm, Bertin Technologies) and a Precellys 24 Homogenizer (Bertin Technologies) with 2 × 30 s at 6000 rpm, following three freeze‒thaw cycles before centrifugation (800 × g, 20 min, 4 °C). The supernatants were collected and stored at −80 °C until analysis. Blood samples underwent three freeze‒thaw cycles and were diluted 1:2 in HEPES-Hanks (Sigma‒Aldrich) buffer supplemented with 0.6% FBS before titration. Replicating virus was quantified using the TCID₅₀ method.

### Antigen-specific antibodies and neutralizing antibody assays

IgG, IgG1, and IgG2a antibodies were quantified using an indirect ELISA following previously established protocols [48]. To assess vaccine antigen-specific responses (i.e. antibodies directed against the same antigen that each mouse received during immunization), ELISA plates were coated with 50 ng per well of the corresponding antigen (Formalin inactivated CVB1, CVB3, CVB6, Full VLP, or VLPΔpalxa), diluted in PBS (pH 7.4). Serum antibody binding was detected via the following HRP-conjugated anti-mouse secondary antibodies: IgG (1:3000), IgG1 (1:1000), and IgG2a (1:1000) (Invitrogen, USA). The endpoint titre was determined as the highest dilution at which the optical density (OD) exceeded the experimental cut-off value. For consistency across experiments, the positivity threshold was set at half of the initial serum dilution (1:200). Antibody titres were plotted as geometric mean titres (GMTs) for each experimental group.

Additionally, neutralizing antibody responses against live viruses were evaluated using a micro-neutralization assay based on cell viability in GMK cells, as described by Soppela et al. [40]. Briefly, serial twofold dilutions of sera were incubated with 50 × TCID_50_ of CVB1 for 1 h at 37 °C, followed by the addition of GMK cells. After 46 h of incubation, cell viability was determined using alamarBlue reagent. Fluorescence was measured as described for the TCID_50_ assay. Neutralization curves were plotted using GraphPad Prism 9.0.0 with a 4-PL curve fit, and the IC_50_ values were determined from the midpoint of the sample-specific neutralization curve. Geometric mean IC_50_ titres (± geometric standard deviation) were used to quantify the neutralizing antibody responses.

### Viral titration by TCID50 assay

Replicating virus in pancreas homogenates and blood samples was quantified using the TCID₅₀ method as previously described [40]. In brief, serial sixfold dilutions of each sample were prepared in 96-well plates containing 16 000 GMK cells/well in MEM with 5% FBS. After 46 h of incubation at 37 °C, viral cytopathic effects were assessed via an alamarBlue viability reagent (Invitrogen). The fluorescence was measured at 560 nm/590 nm with an ENVISION UV/VIS plate reader (Perkin Elmer), and the TCID_50_ calculation was based on Karber’s formula [49].

#### Statistical analyses

GraphPad Prism 9.0.0 was used for the statistical analyses. The threshold for a significant difference was defined as a *p* value < 0.05. The Kruskal‒Wallis test followed by Dunn’s multiple comparison test was used to define differences in the end-point titres between experimental groups, except for comparisons between 2 and 3 doses, which were evaluated with the Mann‒Whitney test.

## Results

### PALXA region-specific antibodies are induced after immunization with inactivated CVBs

To investigate whether formalin-inactivated CVBs induce antibodies targeting the conserved PALXA region of the VP1 N-terminus, three BALB/c mice were immunized sequentially with formalin-inactivated CVB1, CVB3, and CVB6, since all CVBs share a homologous PALXA motif. The mice received 12 µg of each vaccine without adjuvant at three-week intervals and were sacrificed at week 9.

**Fig. 1:**
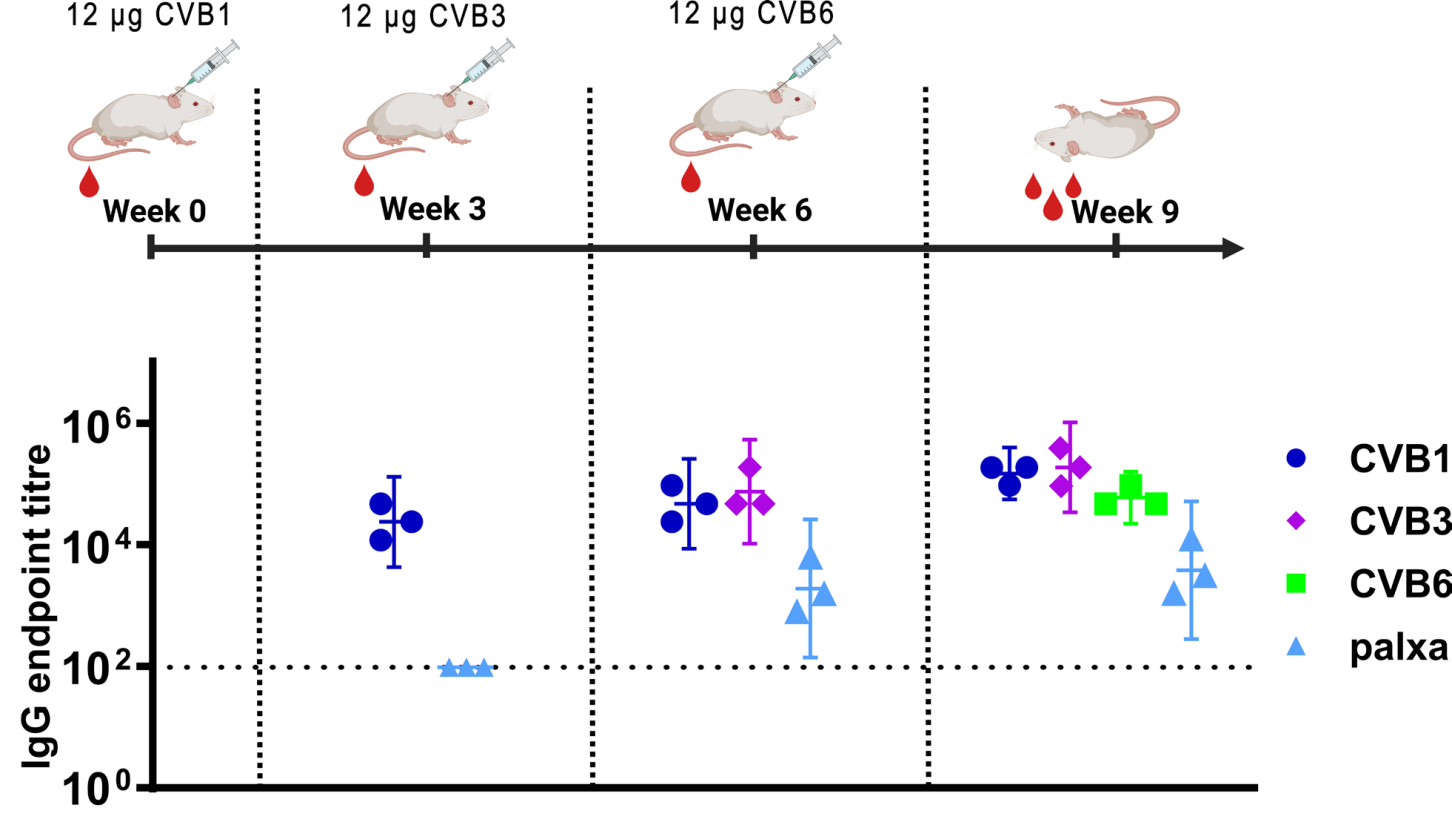
Sequential immunization of mice with formalin-inactivated Coxsackievirus B vaccines induces antibodies against the non-neutralizing PALXA region. Total IgG antibody levels against Coxsackie B 1, −3 and −6 viruses (CVB1/−3/−6) and against SESIPALTAAETGHT-peptide (designated PALXA peptide). BALB/c female mice (n = 3) were immunized serially with 12 µg of formalin-inactivated virus, first with CVB1 (wk 0), then with CVB3 (wk 3) and finally with CVB6 (wk 6). Vaccine-specific and PALXA-peptide-specific IgG-antibody levels were analyzed in sera collected from mice on week 0/3/6/9 (week 0 sera negative, not shown). Titres below 200 were assigned as negative. Shown as scatter dot plots with geometric mean titre and 95% confidence interval

As shown in Fig. 1, blood samples collected between the immunizations indicated that a single vaccine dose elicited some CVB capsid-specific antibodies; however, PALXA region-specific antibodies remained undetectable after a single CVB immunization (with CVB1). When the mice had been immunized with CVB1 (week 0) and with CVB3 (week 3), CVB1- and CVB3-specific antibody levels were comparable by week 6, and PALXA-specific antibodies were detectable, suggesting that in CVB-naïve mice, two immunizations were necessary to trigger a response against the PALXA region. The mice were further immunized with inactivated CVB6 at week 6, and by week 9, CVB1-, CVB3-, and CVB6-specific antibody levels were similar, while PALXA-specific antibody levels appeared to plateau, showing no substantial increase compared with those at week 6.

This finding shows that antibody responses against the conserved VP1 PALXA region were increased in naïve mice after two immunizations with inactivated whole-virus enterovirus vaccines, even when the vaccinations were done with different serotypes.

### The VLPΔpalxa vaccine is highly pure and the particles assemble similarly to unmodified CVB1-VLP

**Fig. 2:**
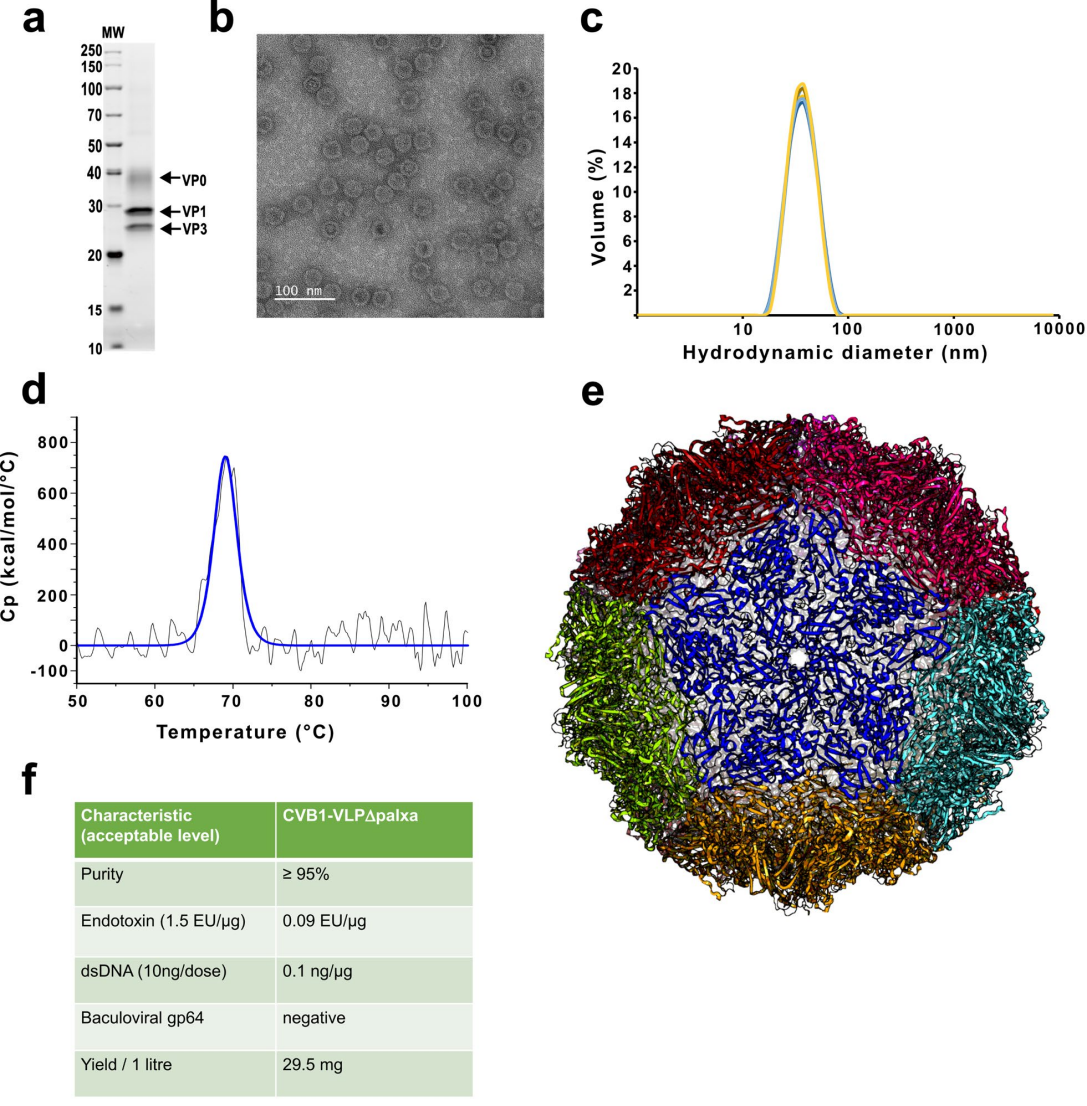
Characterization of purified CVB1-VLPΔpalxa. **a** SDS‒PAGE analysis and stain-free total protein staining of purified VLPs (capsid proteins are visualized with arrows). **b** Transmission electron micrograph of negatively stained VLPs. Magnification 60,000x, Scale bar 100 nm. **c** Dynamic light scattering analysis of the purified particles, including 6 consecutive measurements. **d** Differential scanning calorimetry analysis of the purified particles. **e** Crystal structure of VLPΔpalxa showing an intact icosahedral particle (PDB code 9SCW). Individual pentamers are colored distinctly to visualize their arrangement in the capsid. **f** Characteristics of the purified particles, including the purity of the CVB1-VLPΔpalxa stock; its endotoxin content; dsDNA and baculovirus contents; and total yield of the pure VLP

VLPΔpalxa was produced and purified using previously established methods [43], and the VLPs were characterized via several methods (Fig. 2). SDS‒PAGE and total protein staining of the capsid proteins confirmed the presence of proteins of approximately 38 kDa, 29.5 kDa and 26 kDa corresponding to the expected molecular sizes of VP0, VP1 devoid of 15 amino acids from the N-terminus, and VP3, respectively (Fig. 2a, Supplementary Fig. S1). Densitometric analysis of the proportional amounts of the proteins revealed that the purity of the VLPs was ≥ 95%. Transmission electron microscopy verified that intact particles with the expected spherical capsid morphology were produced (Fig. 2b), and the VLPs closely resembled the size and morphology of the native CVB1 virus we produced previously [50].

Dynamic light scattering analysis revealed that 100% of the particles had an average hydrodynamic diameter of 27.6 nm (± 0.255) with a polydispersity index of 0.09 (± 0.004) (Fig. 2c), demonstrating that a homogeneous particle population corresponding to the size of the native CVB1 virus and unmodified CVB1-VLP could be produced, as we have previously shown [40].

To assess whether modifying the VLP affects the stability of the particle, thermal stability analysis was performed via differential scanning calorimetry (DSC). The melting temperature (Tm) of VLPΔpalxa was 69.05 °C ± 0.05 (Fig. 2d), which is comparable to that of unmodified VLP (Tm 69.78 °C ± 0.09) [43], indicating that the modification did not compromise the thermostability of the particles. To further confirm the structural integrity of VLPΔpalxa, we determined the crystal structure of the particle with 3.2 Å resolution (PDB code 9SCW). VLPΔpalxa formed similar particles to the unmodified CVB1-VLP (PDB code 9FJC [40]) confirming the correct assembly of VLPΔpalxa (Fig. 2e, Supplementary Table S1).

The results of the quality analyses and structural determination revealed that the 15 amino acid deletion from VLPΔpalxa did not prevent particle formation. Notably, the yield of VLPΔpalxa was 29.5 mg/l (Fig. 2f), which was 2.5 times higher than the yield of the previously produced unmodified VLP [40], indicating that the particle stability may have increased compared with that of the unmodified VLP. Additional quality assessments of the vaccine stocks confirmed the absence of residual contaminants from the production process. Baculoviral gp64 proteins were not detected in the preparation; the residual dsDNA level was 0.1 ng/µg (acceptable limit is 10 µg/dose [51]), and the endotoxin level was 0.09 EU/µg (acceptable limit is 1.5 EU/µg [52]) (Fig. 2f). As such, these analyses confirmed that the vaccine stock is free of baculoviral gp64 proteins, with residual dsDNA and endotoxin levels that fit well within acceptable limits.

### VLPΔpalxa, unmodified VLP and formalin-inactivated CVB1 vaccines are well tolerated and demonstrate clear dose–response relationship

**Fig. 3:**
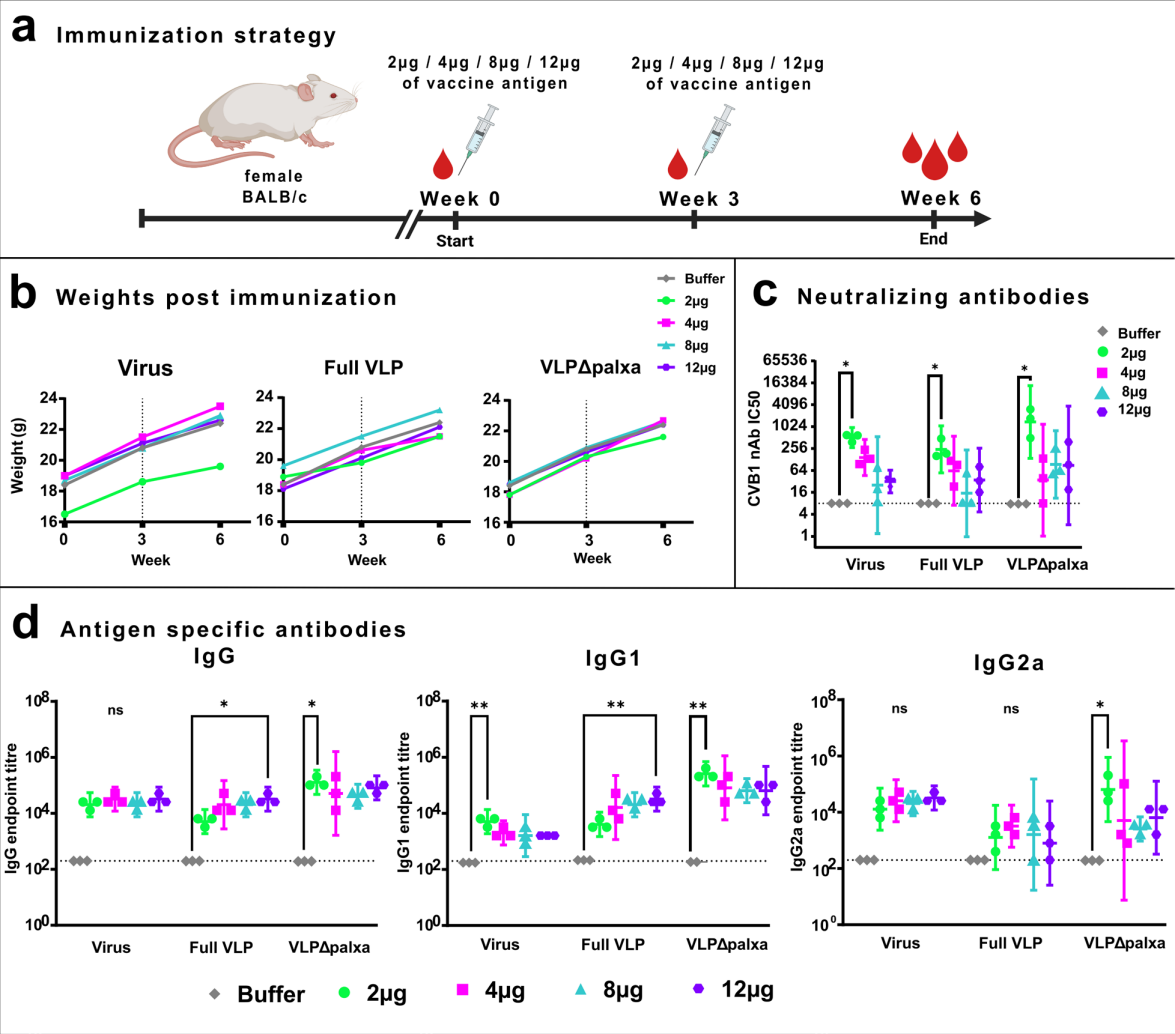
Assessing the safety and determining the optimal dose for the non-adjuvanted CVB1 vaccines. **a** Immunization strategy, Balb/c female mice (n = 3 per group) were immunized with different doses of vaccine candidates to determine the optimal dose. **b** Average weight of three animals for each dose group in three experimental groups. Dashed line indicates the booster vaccination (vaccinations at week 0 and 3). **c** CVB1-neutralizing antibody (nAb) end-point titres determined from termination serum analyzed with a microneutralization assay. Antibody titres less than 8 were assigned as negative (indicated by a horizontal line). **d** Vaccine antigen-specific IgG, IgG1, and IgG2a antibody end-point titres at week 6, determined with indirect ELISA. Titres below 200 were assigned as negative. Shown as scatter dot plots with geometric mean titre and 95% confidence interval. *: *p* < 0.05, **: *p* < 0.01

To study the immunogenicity and safety of the CVB1-VLPΔpalxa vaccine, we performed studies in mice in which we also included formalin-inactivated CVB1 virus and unmodified CVB1-VLP (hereon designated Full VLP) that were produced and analyzed in previous [40] murine experiments. The vaccines used in the experiments are designated CVB1 vaccines hereon. First, the optimal CVB1 vaccine dose was evaluated in a study in which the mice received two doses of 2, 4, 8, or 12 µg of vaccine with a three-week interval (weeks 0 and 3) between doses. The animals were then sacrificed at week 6 (Fig. 3a). All the mice maintained normal weight gain and showed no signs of adverse effects, as well as good overall well-being indicating that the vaccines were safe (Fig. 3b).

High levels of neutralizing antibodies have been shown to protect against enterovirus infections [42]. Therefore, we considered neutralizing antibody titres as the primary parameter for selecting the optimal CVB1 vaccine dose in our further immunization experiments. When the doses within each vaccine group were compared, 2 µg elicited the highest neutralizing antibody titres across all vaccine candidates (Fig. 3c), with geometric mean titres (GMTs) of 512.4 for the formalin-inactivated virus group (*p* = 0.0148), 243 for the Full VLP group (*p* = 0.0216), and 1376 for the VLPΔpalxa group (*p* = 0.0148).

When vaccine antigen-specific total IgG, IgG1 and IgG2a antibody levels were assessed from the vaccinated mice, formalin-inactivated CVB1 showed no significant differences in IgG and IgG2a levels between the groups that received different antigen doses, but for IgG1, the 2 µg dose appeared to elicit the highest response (*p* = 0.0087) (Fig. 3d). In contrast, Full VLP showed the highest IgG and IgG1 levels at the 12 µg dose (*p* = 0.0341 and *p* = 0.0347, respectively), while IgG2a levels remained comparable across the different doses (Fig. 3d). In the VLPΔpalxa group, all antibody levels were highest after the 2 µg dose, with statistically significant differences observed for IgG (*p* = 0.0326), IgG1 (*p* = 0.0114), and IgG2a (*p* = 0.0174) (Fig. 3d). Based on these results, 2 µg of vaccine was determined to be the optimal CVB1 vaccine dose.

#### 2 µgs of non-adjuvanted CVB1 vaccines elicit high humoral responses after two immunizations

**Fig. 4:**
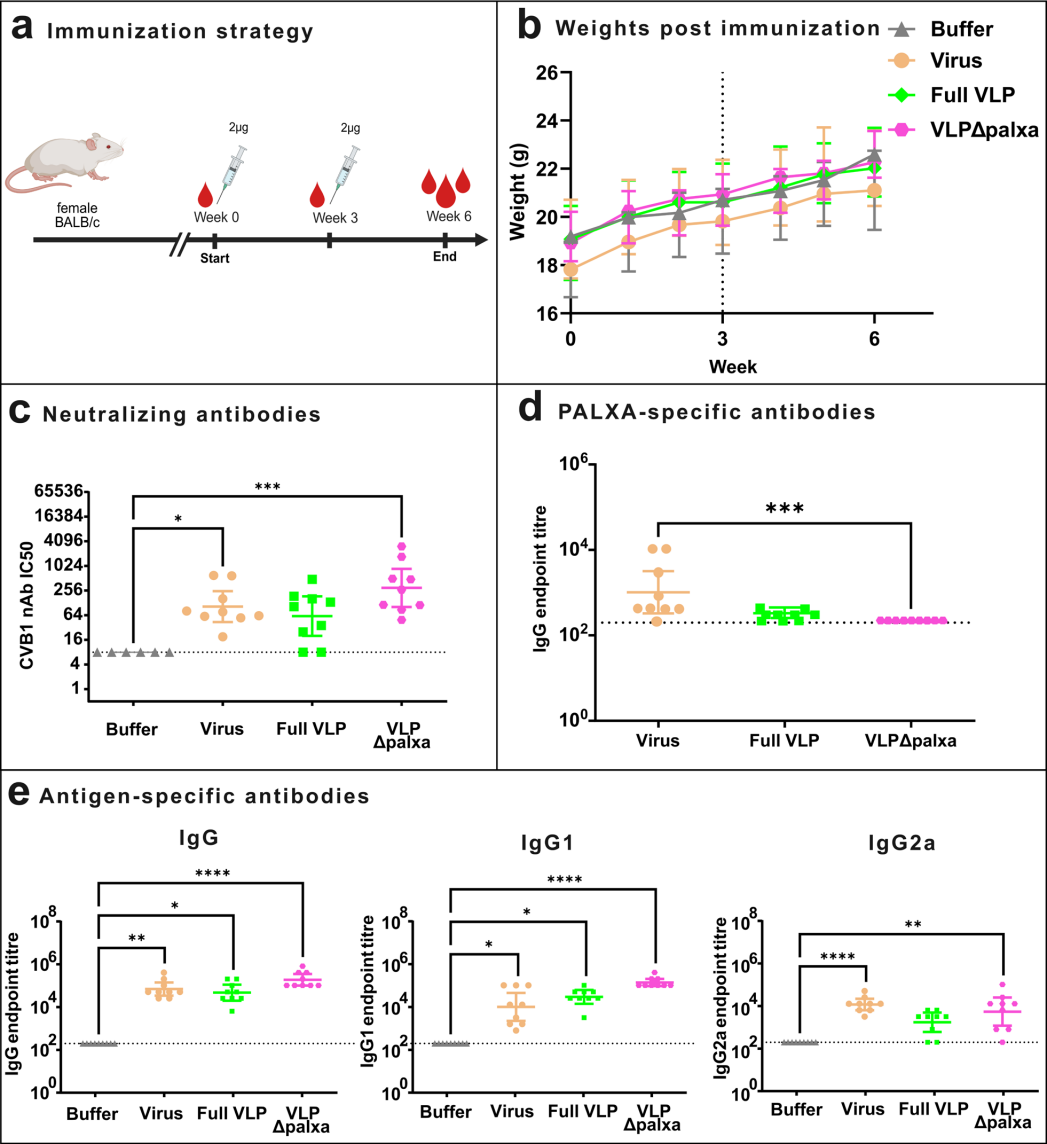
A 2 µg vaccine dose with non-adjuvanted CVB1 vaccines elicit high humoral responses in mice after two vaccine doses. **a** Immunization strategy, Balb/c female mice (n = 9 per group) were immunized twice with a 2 µg vaccine dose without adjuvant. **b** Average weight of 9 animals per experimental group. Dashed line indicates the booster vaccination (vaccination on week 0 and 3). **c** CVB1-neutralizing antibody end-point titres determined from termination serum with a microneutralization assay. Antibody titres below 8 were assigned as negative. **d** PALXA-peptide -specific IgG antibodies determined with peptide-ELISA. **e** Vaccine antigen-specific IgG, IgG1, and IgG2a antibody end-point titres at week 6 determined with indirect ELISA. Titres below 200 were assigned as negative (indicated by a horizontal line). Shown as scatter dot plots with the geometric mean titre and 95% confidence interval. *: *p* < 0.05, **: *p* < 0.01, ***: *p* < 0.001, **** *p* < 0.0001

Based on the vaccine dose titration study findings (Fig. 3), the 2 µg vaccine dose was selected as the optimal immunization dose, and a follow-up study was conducted with CVB1-VLP vaccines with a larger group size (n = 9). BALB/c mice were immunized twice at three-week intervals and sacrificed at week 6 (Fig. 4a). While the dose titration study included a lower number of weight measurements, additional time points were added in this study to ensure that vaccination did not cause weight loss at any time point. These measurements showed that the mice maintained stable weight gain throughout the study (Fig. 4b), demonstrating that the vaccine was well tolerated.

Neutralizing antibody responses were highest in the VLPΔpalxa group, with a GMT of 298 (*p* = 0.0003), followed by the formalin-inactivated virus group, with a GMT of 103.7 (*p* = 0.0143). No significant difference was observed between the Full VLP and Buffer groups, with the Full VLP GMT of 60.8 (Fig. 4c). To assess immune responses against the PALXA epitope, sera were screened against PALXA-peptide-specific IgG antibodies. As expected, the VLPΔpalxa-vaccinated group was negative for antibodies recognizing this epitope, whereas only the formalin-inactivated virus group elicited a significant response toward this peptide, with a GMT of 1089 (*p* = 0.004) (Fig. 4d).

As shown in Fig. 4e, compared with the buffer group, all the vaccine groups presented significant levels of vaccine-specific IgG antibody responses (*p* = 0.0031 for the formalin-inactivated virus group, *p* = 0.0117 for the Full VLP group, and *p* < 0.0001 for the VLPΔpalxa group) and IgG1 responses (*p* = 0.0212 for the formalin-inactivated virus group, *p* = 0.0053 for the Full VLP group, and *p* < 0.0001 for the VLPΔpalxa group). However, the IgG2a responses were not significantly different in the Full VLP group compared to controls, whereas they were robust in the formalin-inactivated virus (*p* < 0.0001) and VLPΔpalxa (*p* = 0.0011) groups.

In short, the VLPΔpalxa group presented the highest neutralizing, IgG, and IgG1 titres, while the formalin-inactivated virus group generated the highest IgG2a response. Additionally, the VLPΔpalxa-specific IgG2a level was statistically significant compared to the buffer group, indicating that the formalin-inactivated virus and VLPΔpalxa may produce a more balanced Th1/Th2 response than the Full VLP.

### Three non-adjuvanted CVB1 vaccine doses induce higher neutralizing antibody levels than two doses

**Fig. 5:**
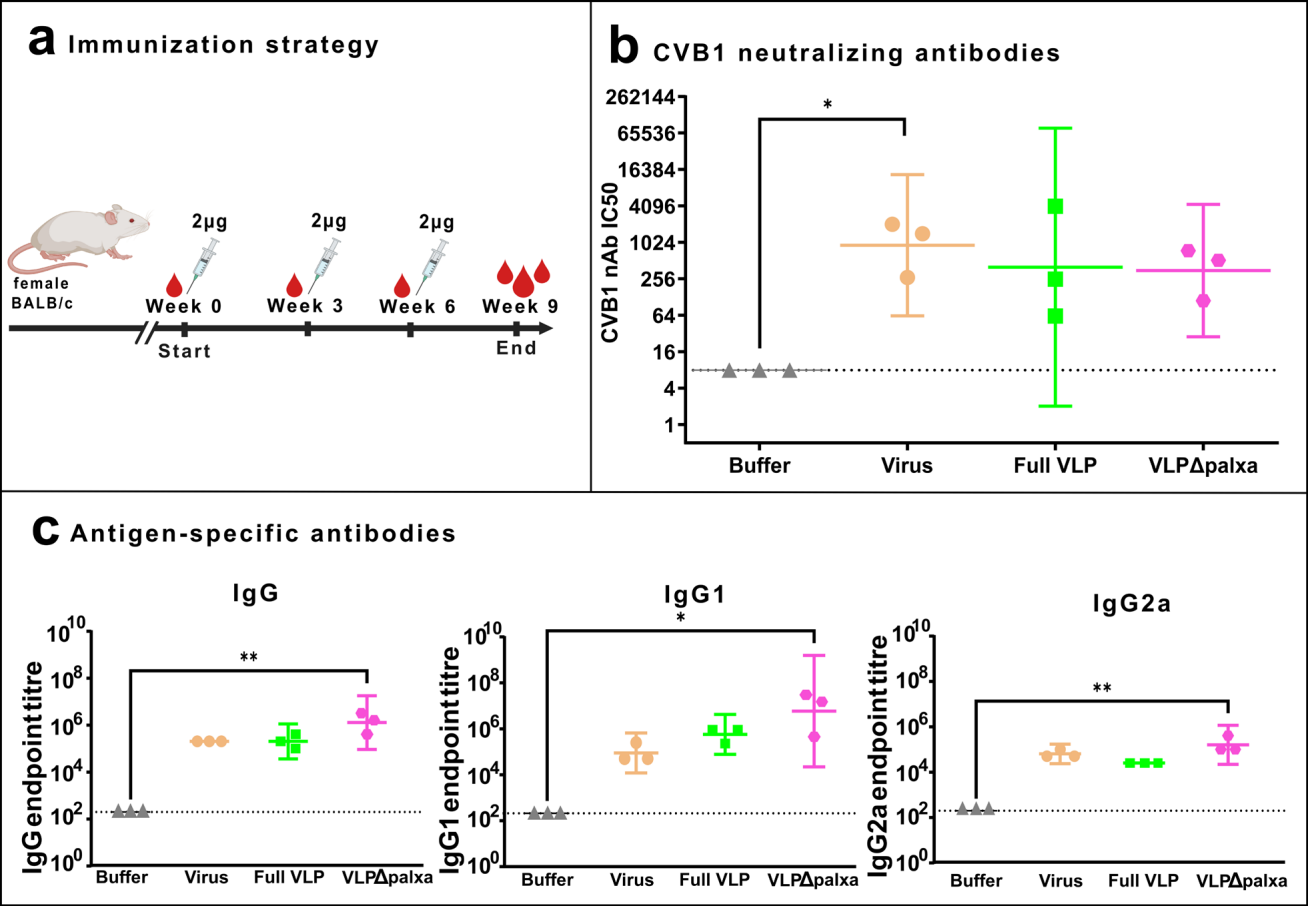
Three non-adjuvanted CVB1 vaccine doses induce high antibody levels. **a** Immunization strategy, Balb/c female mice (n = 3 per group) were immunized with a 2 µg dose three times. **b** CVB1-neutralizing antibody end-point titres determined from termination serum with a microneutralization assay. Antibody titres below 8 were assigned as negative (indicated by a horizontal line). **c** Vaccine antigen-specific IgG, IgG1, and IgG2a antibody end-point titres at week 6 determined with indirect ELISA. Titres below 200 were assigned as negative. Shown as scatter dot plots with geometric mean titre and 95% confidence interval. *: *p* < 0.05, **: *p* < 0.01

To evaluate the levels of neutralizing antibodies and antigen-specific IgG, IgG1 and Ig2a antibodies induced after two or three vaccine doses, we performed a small-scale study in which three mice per group received three vaccine doses (a low number of mice was justified with ethical considerations) (Fig. 5a).

Neutralizing antibody GMTs increased in all groups following the three immunizations (Table 1). The VLPΔpalxa group showed the highest neutralizing antibody titre after two doses (GMT 298) compared to other groups, and the neutralizing antibody GMT increased following a third immunization to 350.6 (*p* = 0.7273) (Table 1 and Fig. 5b). In contrast, the formalin-inactivated virus group presented a significant increase in neutralizing antibody titres, increasing from 103.7 after two doses to 921.5 after three doses (*p* = 0.0364). A similar trend was observed in the Full VLP group, where titres increased from 60.8 to 398.7, although the result remained statistically insignificant (*p* = 0.1955).

**Table 1:** Comparison of neutralizing and IgG antibody titres between two and three immunizations. Geometric mean titres of neutralizing antibodies and vaccine-specific IgG antibodies from BALB/c mice immunized two or three times with non-adjuvanted CVB1 vaccines. P values demonstrate whether or not the third vaccine dose significantly increased the antibody titres. *p < 0.05

Vaccine group	Neutralizing antibody GMT			IgG antibody GMT		
	2 doses	3 doses	*P* value	2 doses	3 doses	*P* value
Inact. virus	103.7	921.5	*	69,672	204,800	–
Full VLP	60.8	398.7	–	47,405	204,800	–
VLPΔpalxa	298	350.6	–	189,619	1,300,399	*

Quantification of antigen-specific antibody responses revealed that total IgG GMTs increased in all groups after three immunizations (Table 1 and Fig. 5c). In the formalin-inactivated virus group, the IgG GMTs increased from 69,672 after two doses to 204,800 after three doses. A similar pattern was observed in the Full VLP group (47,405 to 204,800) and the VLPΔpalxa group, which showed a more pronounced increase from 189,619 to 1,300,399 (*p* = 0.0182).

For IgG1 (Table 2 and Fig. 5c), GMTs in the formalin-inactivated virus group increased from 10,159 to 81,275, whereas those in the Full VLP group significantly increased from 29,863 to 516,064 (*p* = 0.0045). The VLPΔpalxa group demonstrated the highest IgG1 response, with GMTs increasing from 139,345 to 5,201,596 (*p* = 0.0091).

**Table 2:** Comparison of IgG1 and IgG2a antibody titres between two and three immunizations. Geometric mean titres of vaccine-specific IgG1 and IgG2a antibodies from BALB/c mice immunized two or three times with non-adjuvanted CVB1 vaccines. P values demonstrate whether the third vaccine dose significantly increased the antibody titres. *p < 0.05, **p < 0.01

Vaccine group	IgG1 antibody GMT			IgG2a antibody GMT		
	2 doses	3 doses	*P* value	2 doses	3 doses	*P* value
Inact. virus	10,159	81,275	–	11,851	64,508	*
Full VLP	29,863	516,064	**	1728	25,600	**
VLPΔpalxa	139,345	5,201,596	**	5486	162,550	*

Similarly, IgG2a titres increased across all groups after three immunizations (Table 2 and Fig. 5c). In the formalin-inactivated virus group, the IgG2a GMTs increased from 11,851 to 64,508 (*p* = 0.0182), whereas those in the Full VLP group increased from 1728 to 25,600 (*p* = 0.0091). The VLPΔpalxa group demonstrated the highest IgG2a response, with titres increasing from 5486 to 162,550 (*p* = 0.0182).

These results suggest that increasing the number of immunizations enhances both IgG1 and IgG2a responses, particularly in the VLPΔpalxa group, where a significant increase in antibody titres was observed, but there was no notable increase in the neutralizing antibody titres in this group.

### Non-adjuvanted CVB1 vaccines protect mice against viremia

**Fig. 6:**
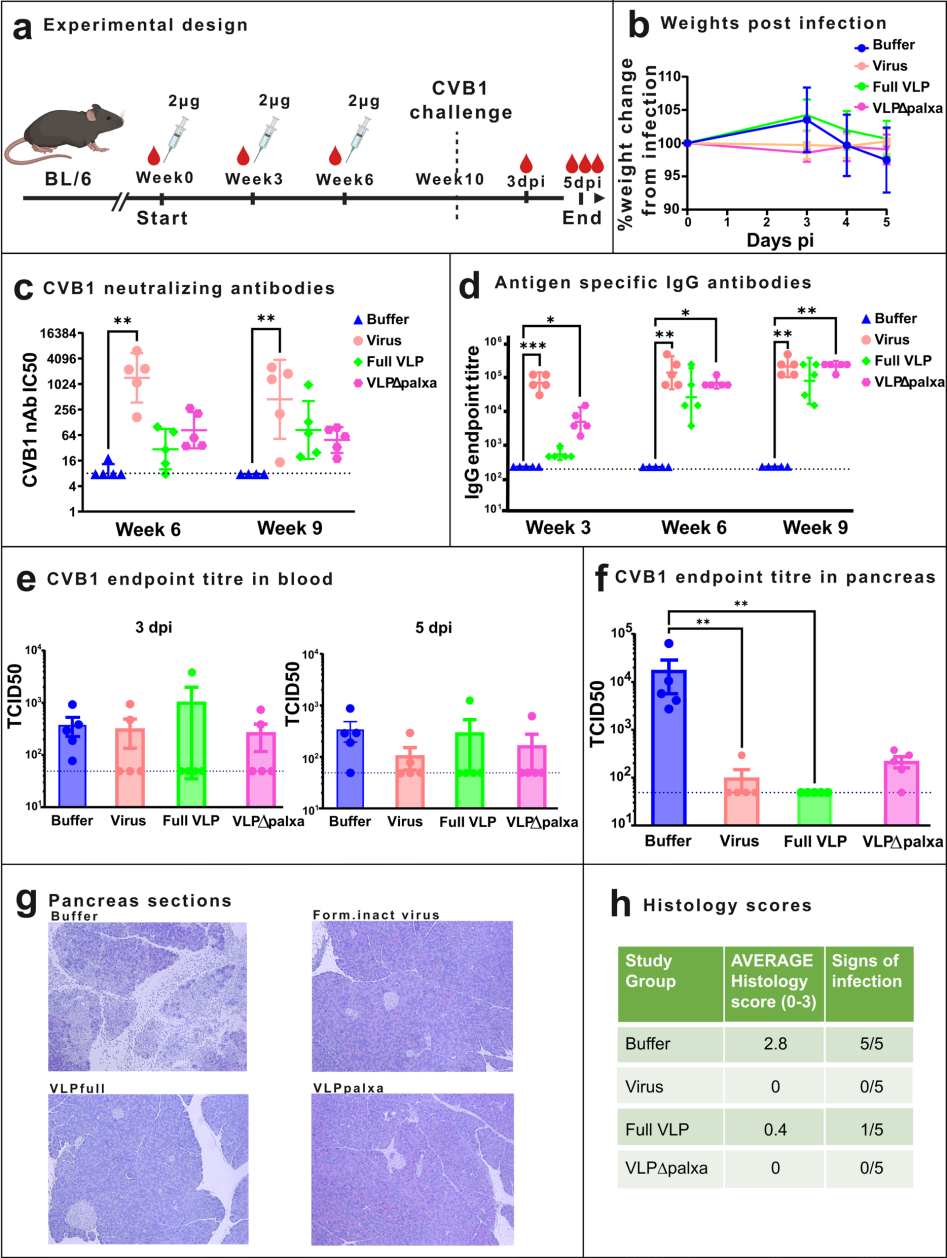
Non-adjuvanted CVB1 vaccines protect mice against viremia. **a** Experimental design, C57Bl/6J mice (n = 5 per group) were immunized with a 2 µg dose three times. **b** Animal weights post infection. **c** CVB1-neutralizing antibody end-point titres determined from Week 6 and Week 9 sera with microneutralization assay. Antibody titres below 8 were assigned as negative (indicated by a horizontal line). **d** Vaccine antigen-specific IgG end-point titres at week 3, 6 and 9, determined with indirect ELISA. Titres below 200 were assigned as negative. Shown as scatter dot plots with Geometric mean and 95% confidence interval. **e** Viral titration by TCID_50_ assay from blood samples 3- and 5-days post infection (dpi) and **f** pancreas 5dpi. The column shows the mean values with SEM and individual scatter dots represent individual mice. Detection limit 49 TCID_50_ units/ml has been indicated by a horizontal line. **g** Representative histological images of pancreatic tissue from each study group. **h** Histology scores based on pancreatic sections. *: *p* < 0.05, **: *p* < 0.01, ***: *p* < 0.001

Next, we evaluated the immunogenicity of the vaccine candidates in mice with both a different genetic background and gender and we also examined whether vaccinations protected mice from a virus challenge. C57Bl/6J (BL/6) mice were immunized with 2 µg dose of non-adjuvanted vaccines on three occasions and were then challenged with infective CVB1 at week 10 post initial vaccination (Fig. 6a). Following infection, average body weights in the buffer group declined notably by day 3 post-infection (likely due to the productive virus infection which made the animals feel sick and eat less), whereas mice in the formalin-inactivated virus and VLPΔpalxa vaccinated groups maintained their weights. The Full VLP group showed a moderate weight loss, though not as pronounced as in the buffer group (Fig. 6b).

As shown in Fig. 6c, neutralizing antibody titres were highest in the formalin-inactivated virus group at week 6 and week 9. One week before the challenge (week 9), GMTs were 409 in the formalin-inactivated virus group (*p* = 0.0032), 81.8 in the Full VLP group, and 48.2 in the VLPΔpalxa group.

Antigen-specific IgG levels increased progressively in all groups after each immunization, as expected, although antibody responses in the Full VLP group appeared to have a delayed onset compared with those in the other vaccine groups (Fig. 6d). At week 3, both the formalin-inactivated virus group (*p* = 0.0001) and the VLPΔpalxa group (*p* = 0.0204) presented significantly higher IgG levels compared to the buffer group. This trend continued at week 6, with significant differences seen between the formalin-inactivated virus group and the buffer group (*p* = 0.0013) as well as between the VLPΔpalxa group and the buffer group (*p* = 0.0191). By the final time point at week 9, the IgG levels remained significantly elevated in both the formalin-inactivated virus (*p* = 0.0063) and VLPΔpalxa (*p* = 0.0048) groups compared with those in the buffer control group, indicating a robust and sustained antibody response in these groups. In contrast, due to the delayed onset of antibody titres and greater variance in the Full VLP group, this difference did not reach statistical significance at any time point.

Upon viral challenge, the viral loads of the replicative virus in the pancreas were significantly lower in the virus and Full VLP groups than in the buffer control group (Fig. 6f). The mean TCID_50_ values were 17,324 in the buffer group, whereas inactivated-virus vaccinated mice showed a substantial reduction to 98 (*p* = 0.005), and the Full VLP vaccinated mice to 49 (*p* = 0.0011). In the VLPΔpalxa group, viral loads were also decreased (mean TCID_50_ = 217.3), but the reduction was not statistically significant. No significant differences in replicative virus levels were observed in blood samples collected at 3 and 5 days post-infection (Fig. 6e).

Histological analysis of pancreatic tissues revealed no signs of infection in the virus and VLPΔpalxa vaccine groups. In contrast, 1 out of 5 mice in the Full VLP group showed some signs of infection, whereas all 5 mice in the buffer group had significantly higher histological scores, which are indicative of pancreatic infection. Signs of infection included destruction of exocrine tissue and infiltration of immune cells (Fig. 6g).

### CVB1 vaccines formulated with adjuvant system 04 induce potent antibody responses in mice

Prior to the challenge study, all the animal experiments were conducted using BALB/c mice, where the VLPΔpalxa vaccine demonstrated a strong ability to induce neutralizing antibodies. However, in the challenge study performed in BL/6 mice, the formalin-inactivated virus group induced the highest neutralizing antibody titres, surpassing both the VLPΔpalxa and Full VLP. Given these findings, we aimed to further optimize the immune response elicited by VLP vaccines. Since currently licensed VLP vaccines, as well as many other vaccines in clinical use, incorporate adjuvants to enhance immunogenicity, we evaluated the efficacy of our vaccine candidates in combination with an adjuvant that is included in VLP-based vaccine against human papillomavirus (Cervarix), in this case the adjuvant system 04 (AS04).

**Fig. 7:**
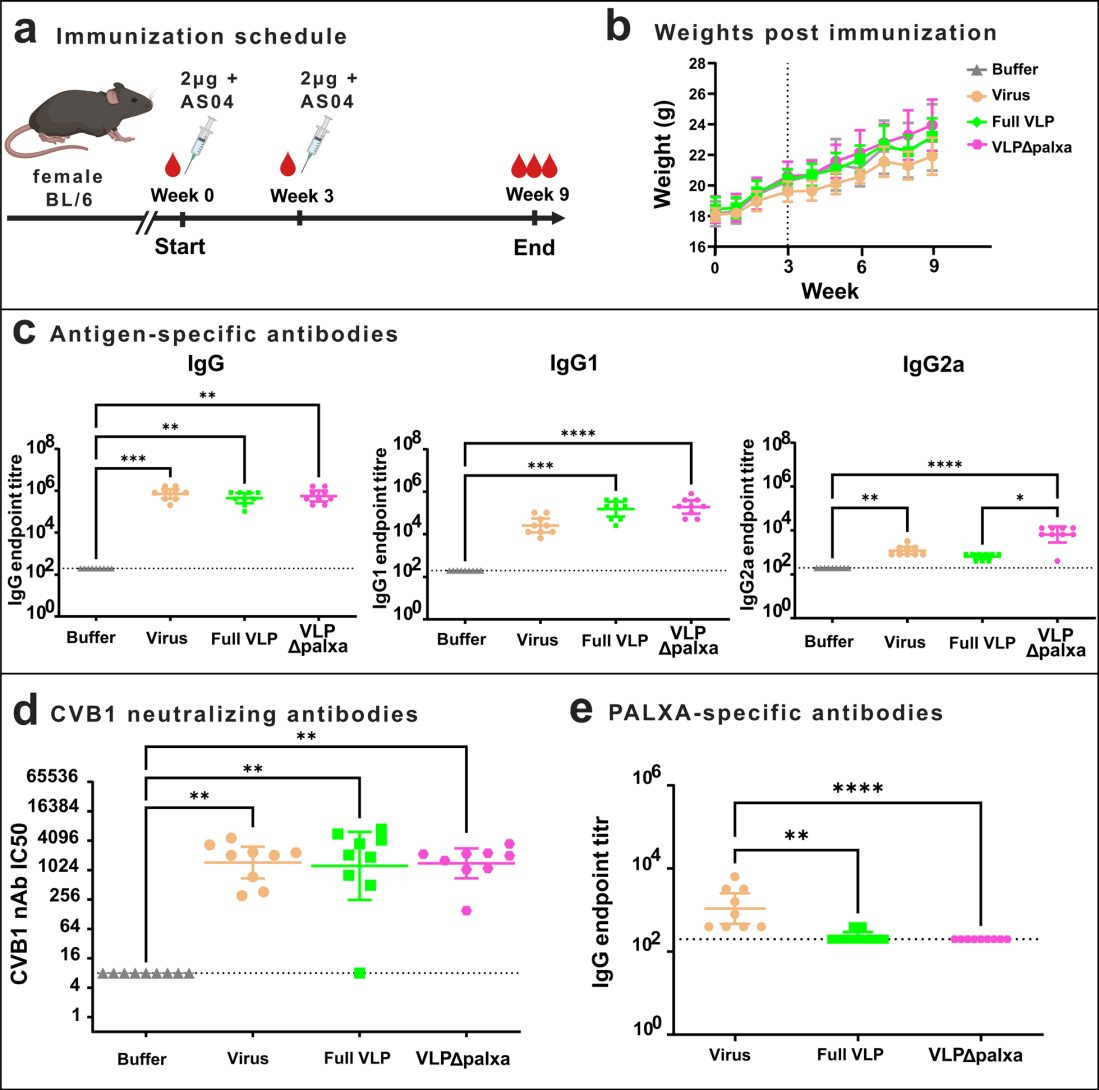
CVB1 vaccines formulated with adjuvant system 04 induce potent antibody responses in mice. **a** Immunization schedule, C57BL/6 J female mice (n = 9 per group) were immunized with a 2 µg dose formulated with adjuvant system 04 (AS04) two times. **b** Average weight of nine animals for each dose group in the experimental groups. Dashed line indicates the booster vaccination (vaccinations at weeks 0 and 3). **c** Vaccine antigen-specific IgG, IgG1, and IgG2a antibody end-point titres at week 9, determined with indirect ELISA. Titres below 200 were assigned as negative. **d** CVB1-neutralizing antibody end-point titres determined from termination serum with a microneutralization assay. Antibody titres below 8 were assigned as negative (indicated by a horizontal line). Shown as scatter dot plots with geometric mean and 95% confidence interval. *: *p* < 0.05, **: *p* < 0.01, ***: *p* < 0.001, **** *p* < 0.0001

The immunization schedule was slightly modified to follow the antibody titres for longer. Nine mice per group received two doses of 2 µg of vaccine antigen formulated with AS04 adjuvant at three-week intervals. Mice were sacrificed at week 9 (Fig. 7a). The mice maintained stable weight gain throughout the study (Fig. 7b), demonstrating that, as with the vaccine alone, the vaccine‒adjuvant combination was well tolerated.

Total IgG levels were significantly elevated in all groups (Fig. 7c), with the formalin-inactivated virus group having the highest antibody titres (*p* < 0.0001 compared with the buffer group), followed by the VLPΔpalxa (*p* = 0.0009 compared with the buffer group) and Full VLP groups (*p* = 0.0025 compared with the buffer group). However, the IgG subclass responses differed among the groups. IgG1 titres were highest in the VLPΔpalxa (*p* < 0.0001 compared with buffer) and Full VLP (*p* < 0.0001 compared with buffer) groups, whereas IgG2a titres were highest in the VLPΔpalxa group (*p* < 0.0001 compared with buffer), followed by the formalin-inactivated virus group (*p* = 0.0010). In contrast, the Full VLP group did not exhibit a significant IgG2a response (*p* > 0.05).

All vaccine groups generated high and comparable levels of neutralizing antibodies (Fig. 7d), with GMTs of 1462 in the formalin-inactivated virus group (*p* = 0.0012 compared with the buffer group), 1251 in the Full VLP group (*p* = 0.0005 compared with the buffer group), and 1412 in the VLPΔpalxa group (*p* = 0.0030 compared with the buffer group). One individual mouse in the Full VLP group did not develop detectable levels of neutralizing antibodies, which created variation in this group. With the adjuvant, neutralizing antibody titres were approximately 3.6-fold (formalin-inactivated virus group), 15.3-fold (VLPΔpalxa group), and 29.3-fold (Full VLP) higher than those in the challenge study (where non-adjuvanted vaccine was used).

Consistent with earlier findings, PALXA-specific antibodies were most prevalent in the formalin-inactivated virus group, and the addition of the adjuvant did not significantly alter this outcome (Fig. 7e). This finding reinforces previous results showing that this immunodominant epitope is preferentially recognized following inactivated virus immunization.

## Discussion

In this study, we evaluated the immunogenicity and protective efficacy of CVB1 vaccine candidates in multiple animal studies. CVB1 was selected due to its heavy burden on healthcare systems (especially with regards to myocarditis) and strong associations with several severe diseases such as T1D. In vaccine production, we utilized the virus and respective sequence based on CVB1 strain 10796 [39, 40]. This virus strain can induce productive infection in mice and we have previously produced a CVB1-VLP based on this strain [40]. This enabled us to compare immune responses elicited by a formalin-inactivated CVB1 vaccine, unmodified CVB1-VLP (Full VLP), and a modified VLP lacking the 15 amino acid-long VP1 N-terminal PALXAXETG motif (VLPΔpalxa). The PALXAXETG motif has been reported to be a 9–25 amino acid-long evolutionarily conserved, antigenic and immunogenic motif in enteroviruses.

The importance of deleting 15 amino acids instead of a shorter stretch was supported by a study conducted by Cello et al. [53], who identified different group-common linear epitopes in EVs and found the PALXA region to be the most reactive among the conserved regions of the VP1‒VP4 capsid proteins. These findings indicated a difference in sensitivity between the peptides PALTAVETGATNPL and PALTAAETG, with the longer sequence showing greater sensitivity. Samuelson [26] determined the key amino acids essential for antibody recognition within this motif to be P, A, L, T, A, E, T, and G, based on substitution peptide analogue studies. Additionally, Roivainen et al. [54] detected the highest antibody titres against an epitope spanning residues 40–53 of Poliovirus type 3/SabinVP1 (PALTAVETGATNPL), whereas Airaksinen et al. [27] found that 25 amino acid long sequence TV**P**A**L**T**A**V**E**T**G**HTSQVTPSDTMQ**TR** in Coxsackie A9 virus (CVA9) VP1 N-terminus includes a 9 amino acid long core motif (underlined), where five amino acids were identical (P, L, A, E, G) and the last two amino acids of the stretch, T and R were also identical among 68 serotypes (79 different sequences) when they uploaded all enterovirus sequences that were available in 1999 in the Picornavirus sequence database [27]. This motif has also been reported as the primary epitope recognized by several monoclonal antibodies that are commonly used in enterovirus detection, further emphasizing the immunoreactivity of this region. IPALTAVETGHT has been determined to be the consensus sequence for the 5D-8.1 [31] epitope and both the 5D-8.1. and 3A6 antibodies recognize the VP1 peptide RPTNSESIPALTAAE [32]. Therefore, we decided to exclude SESIPALTAAETGHT from the CVB1-VLP VP1 N-terminus to avoid the production of immunodominant antibodies by vaccination because these antibodies do not neutralize the virus.

In the context of vaccine development, scientists often remain passive observers of the B-cell selection processes that dictate epitope targeting. This lack of control can limit the effectiveness of vaccines aimed at inducing broadly neutralizing antibodies. A key hypothesis of this study was that by omitting a known non-neutralizing epitope from the viral capsid, the immune response could be directed more effectively toward neutralizing epitopes. Although the neutralizing antibody titres did not consistently improve across all animal studies, the responses induced by the modified VLP were comparable to those induced by the formalin-inactivated CVB1 vaccine, which is considered the current benchmark. Notably, the 15 amino acid deletion from the PALXA region increased the production yield of the particle by 2.6 times compared with that of the unmodified VLP, which we have produced previously [40]. The improved production yield of the modified VLP enhances the applicability of the VLP at the industrial scale and in clinical use in the future.

To assess how this antigen modification affects the immunogenicity and safety of the vaccine, we conducted studies with different immunization strategies. Our experiments included dose titrations, two- versus three-dose regimens, immunizing mice with different genetic backgrounds (namely, BALB/c and BL/6 mice), and the impact of adjuvants on the immune response. In addition to immunogenicity studies, we conducted a challenge study to evaluate the ability of vaccines to protect against CVB1 infection. Our results indicate that CVB1 vaccines are well tolerated and immunogenic, with 2 µg being the optimal dose for eliciting strong neutralizing antibody responses. This result is consistent with our earlier data, in which we compared 0.3, 1, and 10 µg doses of the wild-type CVB1-VLP vaccine and found no positive correlation between increasing dose and neutralizing antibody levels [43]. In fact, the highest dose (10 µg) resulted in the lowest neutralizing antibody responses, suggesting a potential threshold beyond which antigen overload may dampen the immune response. The present dose-titration study confirms this trend, as the lowest tested dose induced the highest neutralizing response. These results highlight the importance of dose–response studies in identifying the optimal antigen quantity required to elicit optimal immunogenicity with vaccines.

In a challenge study, we used BL/6 mice and thereby complemented and extended the initial immunogenicity studies performed in BALB/c mice. The change in strain revealed differences, especially in neutralizing antibody responses. However, relatively low neutralizing antibody titres were sufficient for protection against infection. At five days post infection, the viral load in the blood had been largely cleared from both the vaccinated mice and the buffer group, but the buffer group had detectable levels of replicative virus in the pancreas (Fig. 6).

The addition of the AS04 adjuvant to the vaccine resulted in a substantial increase in neutralizing antibody titres across all groups, elevating neutralising titres by 15-fold in the VLPΔpalxa group and 29-fold in the Full VLP group (Fig. 7). In contrast, the rise in titres was less pronounced in the formalin-inactivated virus group. A possible explanation for this difference is that the inactivated virus already contains viral RNA, which can act as a potent innate immune stimulant through pattern recognition receptors such as toll-like receptors (TLRs), triggering a strong immune response even in the absence of an adjuvant. Adjuvants can induce non-specific activation of the innate immune system, which enhances the magnitude and quality of the adaptive response. This non-specific immune stimulation is central to the mechanism of action of many adjuvants, including AS04, which combines a TLR4 agonist with aluminium salt to boost both humoral and cellular responses [55]. Our recent study demonstrated that a formalin-inactivated CVB1 vaccine can induce robust humoral, cellular and mucosal responses when administrated via intranasal route without adjuvants, but Full VLP induces only mediocre immune response when administrated intranasally [40]. In the same study we were able to solve the atomic level structures of the formalin-inactivated CVB1 and Full VLP, but the only difference between these structures was that cross-linked amino acids are found in the capsid of the inactivated virus, which also contains RNA, neither of which are found in the VLP vaccines. Therefore, we concluded that inclusion of MPLA TLR4 agonist with the VLPs may enhance the immunogenicity of the VLP vaccines to similar levels as with the inactivated virus [40]. While AS04 is known to support both humoral and cellular immunity, our current study focused on humoral responses. To better address this aspect, we intend to perform more comprehensive immune profiling in future studies to include cellular immune response data.

Clinical findings in acute flaccid myelitis (AFM) patients have demonstrated that antibodies against the PALXAXETG motif have been detected in the sera of  > 560 children suffering from AFM and motor paralysis during enterovirus outbreaks in the U.S. from 2014 to 2019 [33]. Similarly, our mouse immunization experiments with inactivated CVB1, CVB3, and CVB6 demonstrated that antibodies targeting the PALXA region were present after immunizing the mice sequentially with different inactivated Coxsackie B virus serotypes (Fig. 1) and following whole-virus vaccination with the same serotype (Figs. 4d and 7e). Studies by Maccari et al. [31] and Saarinen et al. [32] have demonstrated that antibodies 5D-8.1 and 3A6, whose epitopes completely overlap the PALXA region are not neutralizing. As such, removing this region from vaccine candidates could represent a novel approach for improving immunogenicity and preventing immune responses against non-neutralizing targets. However, further studies are needed to confirm whether the PALXA epitope is indeed associated with disease worsening.

The PALXAXETG motif is located within VP1 alongside key neutralizing epitopes such as the BC loop [56], meaning that the VP1 region contains both neutralizing and non-neutralizing antibody targets. For example, Ashton et al. [57] reported that children who developed early insulin autoantibodies had lower levels of VP1-specific antibodies than controls did. However, their study did not distinguish between specific epitopes within VP1, such as the PALXA motif or known neutralizing regions, making it unclear which targets were responsible for the observed association. To better understand the immune targets and epitopes involved with specific types of immune responses, more detailed epitope-specific analyses are needed, including identification and functional characterization epitopes involved with both neutralizing and non-neutralizing immune responses.

Studies have shown that maintaining neutralizing antibody levels above the protective threshold is important and robust memory CD4 + T-cell responses provide long-term immunity against enteroviruses [58]. Additionally, research on individuals vaccinated against or infected with polio or EV-A71 appears to show that an optimal immune response necessitates a well-balanced integration of humoral, cellular, and mucosal immunity [59]. While IgG antibodies do not always correlate directly with neutralizing capacity, they provide an overall view of the humoral immune response. In particular, the IgG1/IgG2a ratio is commonly used as an indicator of Th1/Th2 polarization [60]. Our previous studies demonstrated that IgG1 and IgG2a antibody levels reflect the status of humoral (Th2-type) and cellular (Th1-type) immune responses induced by vaccines [40, 44, 61]. Here, we demonstrated that based on the IgG1/IgG2a ELISA analyses, the candidate vaccines induced rather balanced Th1/Th2 responses (Figs. 3, 4, 5, 6 and 7).Since neutralizing antibodies have been stated to be the most critical factor in clearing CVB infection [62], this study did not directly assess the cellular immunity. However, further characterization of vaccine-induced cellular responses should still be assessed in the future.

To summarize, we demonstrated that targeted structural modification of the CVB1-VLP capsid can reduce immunodominant antibody responses, especially those directed against the conserved PALXAXETG motif after vaccination. Our findings show that modified VLPs, particularly when combined with adjuvants, can elicit robust and balanced antibody responses. In general, VLPs represent a promising vaccine platform due to their excellent safety profile, structural similarity to native viruses with the ability to be engineered with precision. This allows for the removal of undesirable antigenic regions, as demonstrated in our study. The modified CVB1-VLP did not consistently outperform the formalin-inactivated CVB1 vaccine, but its immunogenicity was comparable, especially when formulated with adjuvants. Together with their improved production yield and safety characteristics, CVB-VLPs are highly viable candidates for future enterovirus vaccine development.

## Conclusions

This study demonstrated the feasibility and benefits of targeted structural modification of enterovirus VLPs. We successfully engineered CVB1-VLPs lacking the conserved PALXAXETG motif without compromising particle formation or structural integrity, as confirmed by high-resolution structural analysis. Importantly, the modified VLPs elicited immune responses comparable to those induced by unmodified VLPs, and the production yield was significantly improved. Modified VLPs lacking the conserved PALXAXETG motif elicited strong and balanced humoral responses, especially when combined with adjuvant, and provided protection in a challenge model, even in the absence of adjuvant. These findings highlight the importance of rational design in VLP-based vaccines, producing efficient and safe next-generation enterovirus vaccines.

## Supplementary Information

Additional file 1

## Data Availability

The datasets used and/or analysed during the current study are available from the corresponding author on reasonable request.

## Abbreviations

CVB: Coxsackie B virus
EV: Enterovirus
VP: Viral protein
T1D: Type 1 diabetes
VLP: Virus-like particle
HPV: Human papilloma virus
ADE: Antibody-dependent enhancement
AFM: Acute flaccid myelitis
TFF: Tangential flow filtration
DLS: Dynamic light scattering
TEM: Transmission electron microscopy
DSC: Differential scanning calorimetry
MEM: Minimum Essential Medium Eagle
OD: Optical density
GMT: Geometric mean titre
TCID₅₀: 50% Tissue culture infectious dose
T_m_: Melting temperature
dpi: Days post infection
AS04: Adjuvant system 04
CVA9: Coxsackie A9 virus
TLR: Toll-like receptor
