# Supplementary material for: Coxsackie B1 virus-like particle vaccine modified to exclude a highly conserved immunoreactive region from the capsid induces potent neutralizing antibodies and protects against infection in mice
Source: J Biomed Sci. 2025 Sep 8;32:86. doi: 10.1186/s12929-025-01183-1 (PMC12418688; doi:10.1186/s12929-025-01183-1)
Supplement: Supplementary file 1 — Additional file 1 [file 12929_2025_1183_MOESM1_ESM.docx]

**Supplementary information**

**Coxsackie B1 virus-like particle vaccine modified to exclude a highly conserved immunoreactive region from the capsid induces potent neutralizing antibodies and protects against infection in mice**

**Saana Soppela, Martín González-Rodríguez, Virginia M Stone, Iiris Mustonen, Niila V V Jouppila, Vili Lampinen, Teemu Haikarainen, Malin Flodström-Tullberg, Ilkka S Junttila and Minna M Hankaniemi***

Correspondence: [minna.hankaniemi@tuni.fi](mailto:minna.hankaniemi@tuni.fi), Virology and Vaccine Immunology, Faculty of Medicine and Health Technology, Tampere University, Tampere, Finland.

**List of supplementary information**

Supplementary figure 1

Supplementary table 1


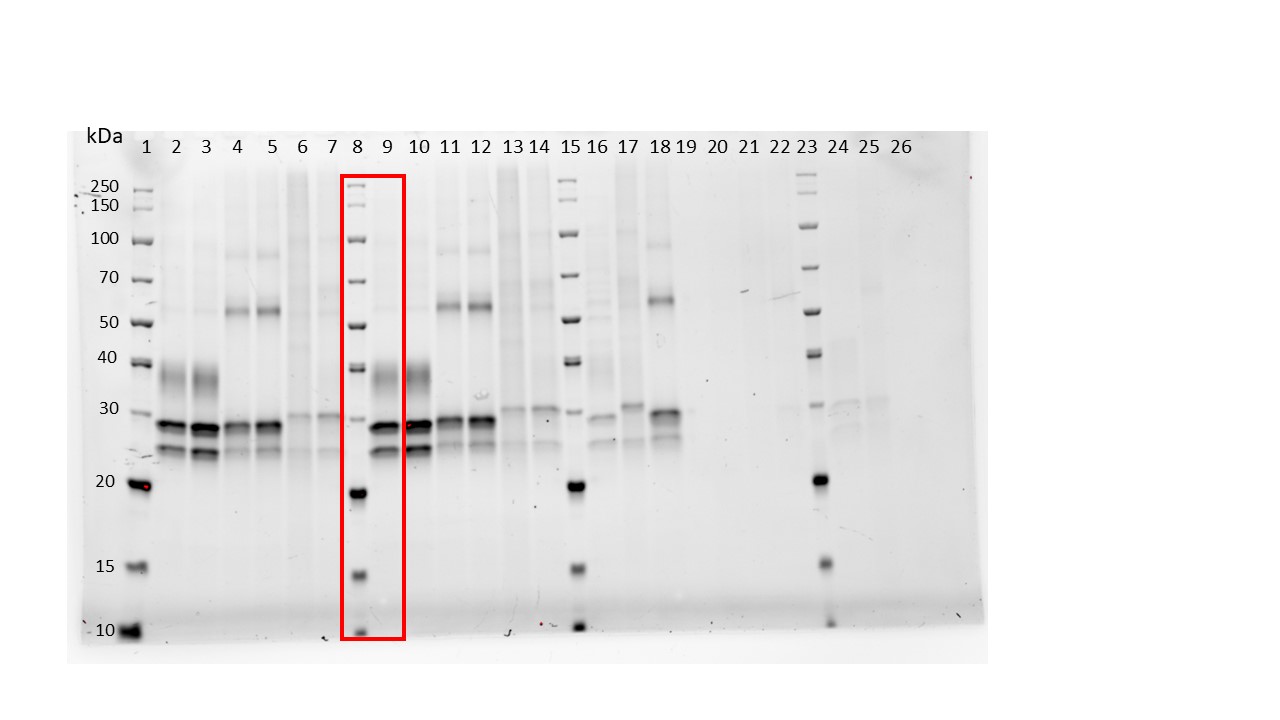


**Supplementary figure 1.** CVB1-VLPpalxa characterization on SDS-PAGE. Full electrophoresis made from the gel were cropped in figure 2. 1) Molecular weight marker, 2-3) CVB1-VLPpalxa 4-7) Irrelevant samples 8) Molecular weight marker 9-10) CVB1-VLPpalxa, 11-14) Irrelevant samples, 15) Molecular weight marker, 16-22) Irrelevant samples, 23) Molecular weight marker, 24-25) Irrelevant samples, 26) empty well. Red boxe indicates the areas cropped for figure 2.

**Supplementary table 1.** Diffraction data collection and refinement statistics.

|  | **CVB1**-**VLPΔpalxa (PDB code 9SCW)** |
| --- | --- |
| **Data** |  |
| Beam line | Diamond I03 |
| Wavelength (Å) | 0.97625 |
| Space group | P312 |
| Cell dimensions |  |
| a, b, c (Å) | 343.99, 343.99, 457.06 |
| α, β, γ (°) | 90.00, 90.00, 120.00 |
| Resolution (Å) | 97-3.20 (3.39-3.20) |
| CC_1/2_ (%) | 94.3 (21.1) |
| *I/σI* | 3.0 (0.3) |
| Completeness (%) | 99.8 (99.3) |
| Redundancy | 8.6 (9.3) |
| **Refinement** |  |
| Reflections | 986596 |
| R_work_/R_free_ | 0.293/ 0.309 |
| RMSD of bond lengths (Å) | 0.003 |
| RMSD of bond angles (°) | 0.6 |
| *B*-factors (Å^2^) |  |
| Protein | 96.94 |

Values for the highest-resolution shell are shown in parentheses.
